# Malaria in migrant agricultural workers in western Ethiopia: entomological assessment of malaria transmission risk

**DOI:** 10.1186/s12936-021-03633-1

**Published:** 2021-02-16

**Authors:** Sisay Dugassa, Mathew Murphy, Sheleme Chibsa, Yehualashet Tadesse, Gedeon Yohannes, Lena M. Lorenz, Hiwot Solomon, Delenasaw Yewhalaw, Seth R. Irish

**Affiliations:** 1 Aklilu Lemma Institute of Pathobiology, Addis Ababa University, Addis Ababa, Ethiopia; 2 Centers for Disease Control and Prevention, 1600 Clifton Road, 30329-4027 Atlanta, GA USA; 3 The US President’s Malaria Initiative, Bureau for Global Health, United States Agency for International Development, 1300 Pennsylvania Ave NW, 20523 Washington, DC USA; 4 U.S. Agency for International Development (USAID), Entoto Street, Addis Ababa, Ethiopia; 5 The President’s Malaria Initiative Private Health Sector Project, Abt Associates Inc, Haile Gebreselassie road, Rebecca Building, 5th Floor, Addis Ababa, Ethiopia; 6 The President’s Malaria Initiative Africa Indoor Residual Spraying Project, Abt Associates, Gerji Road, Sami Building, 1st Floor, Addis Ababa, Ethiopia; 7 Department of Disease Control, London School of Hygiene & Tropical Medicine, Keppel Street, WC1E 7HT London, UK; 8 College of Medicine & Veterinary Medicine, University of Edinburgh, University of Edinburgh, UK; 9 Disease prevention and control directorate, Federal Ministry of Health, Addis Ababa, Ethiopia; 10 Tropical and Infectious Diseases Research Center, Jimma University, Jimma, Ethiopia; 11 Department of Medical Laboratory Sciences and Pathology, College of Health Sciences, Jimma University, Jimma, Ethiopia

**Keywords:** *Anopheles arabiensis*, Malaria, Ethiopia, Agricultural development, Migrant workers

## Abstract

**Background:**

Ethiopia has made great strides in malaria control over the last two decades. However, this progress has not been uniform and one concern has been reported high rates of malaria transmission in large agricultural development areas in western Ethiopia. Improved vector control is one way this transmission might be addressed, but little is known about malaria vectors in this part of the country.

**Methods:**

To better understand the vector species involved in malaria transmission and their behaviour, human landing collections were conducted in Dangur woreda, Benishangul-Gumuz, between July and December 2017. This period encompasses the months with the highest rain and the peak mosquito population. Mosquitoes were identified to species and tested for the presence of *Plasmodium* sporozoites.

**Results:**

The predominant species of the *Anopheles* collected was *Anopheles arabiensis* (1,733; i.e. 61.3 % of the entire *Anopheles*), which was also the only species identified with sporozoites (*Plasmodium falciparum* and *Plasmodium vivax*). *Anopheles arabiensis* was collected as early in the evening as 18:00 h-19:00 h, and host-seeking continued until 5:00 h-6:00 h. Nearly equal numbers were collected indoors and outdoors. The calculated entomological inoculation rate for *An. arabiensis* for the study period was 1.41 infectious bites per month. More *An. arabiensis* were collected inside and outside worker’s shelters than in fields where workers were working at night.

**Conclusions:**

*Anopheles arabiensis* is likely to be the primary vector of malaria in the agricultural development areas studied. High rates of human biting took place inside and outdoor near workers’ residential housing. Improved and targeted vector control in this area might considerably reduce malaria transmission.

## Background

The commitment to eradicate malaria from the globe through increased malaria control and treatment has resulted in a remarkable decrease in the number of cases and mortality associated with this disease [1–3]. It is estimated that the scale-up of the major interventions, long-lasting insecticidal nets (LLINs), indoor residual spraying (IRS) and treatment with artemisinin-based combination therapy reduced malaria cases and mortality by 37 % and 60 %, respectively, between 2000 and 2015 [4]. However, malaria remains a serious disease affecting the well-being of people living in the tropical and subtropical countries of the world and progress against malaria has slowed down considerably in the past few years [1].

In Ethiopia, malaria is still an important cause of morbidity and mortality as in other countries in tropical Africa. Despite a prevalence of less than 1 % in the country, malaria is important in certain foci that pose a risk for epidemics. The National Malaria Control Programme of the Federal Ministry of Health of Ethiopia has recently set an ambitious goal of eliminating malaria from all 565 malarious districts by the year 2030 [5]. In order to achieve this goal, it is important to understand when and where malaria is being transmitted, both at large and small-scale levels. This is important for planning appropriate malaria control strategies and their efficient implementation.

One of the areas where malaria transmission is of concern is in lowland agricultural development areas. Ethiopia has been practicing an Agricultural Development Led Industrialization (ADLI) since 1991 [6]. As a result, various agricultural development areas have been created in the country. Such agricultural investments grow high cash generating crops such as sesame, green grams, cow peas, and sorghum. Agricultural development in such areas has resulted in the migration of hundreds of thousands of seasonal workers into areas where vector-borne diseases, such as malaria and visceral leishmaniasis, are endemic [7].

As these areas offer opportunities for employment, mobile and migrant populations travel to these sites, often during the rainy season for cultivating crops in the area. Those workers stay working in the agricultural development areas until harvesting time. These areas are productive for crops, but also for mosquitoes. This is mainly due to their warm climate and creation of many temporary breeding habitats during the rainy season that provide favourable environment for mosquitoes. Migrant workers may leave mosquito nets in their permanent homes, and stay in temporary, substandard shelters, thus increasing their risk of contracting malaria. Additionally, they may work or remain exposed to mosquitoes in ways and at times that are different from when they are in their hometowns. They may also carry malaria parasites back to their home areas of relatively low malaria risk, complicating the efforts towards malaria elimination in these districts [8].

The biting behaviour of mosquitoes is an important risk factor for infection with malaria parasites [9]. Hence, prevention and control measures for the disease should take the site and time of people’s exposure to mosquito bites into account. There is very little previous research on malaria vectors in Benishangul Gumuz, western Ethiopia, which is home to a large number of agricultural development areas. In order to establish an effective malaria control programme through targeted malaria prevention messages and control interventions, it is essential to understand the mosquito species present in the study area, the venue and times that mosquitoes bite humans and the risk of humans becoming infected with malaria. This study aimed to provide the entomological context of malaria transmission in agricultural settings in this area in tandem with a second study investigating the human behaviour in agricultural development areas. Accordingly, this work aimed to determine the host-seeking behaviour of *Anopheles* mosquitoes in this area that is one of the agricultural development areas in Ethiopia with substantial migrant human populations from highland areas.

## Methods

### Study site

**Fig. 1:**
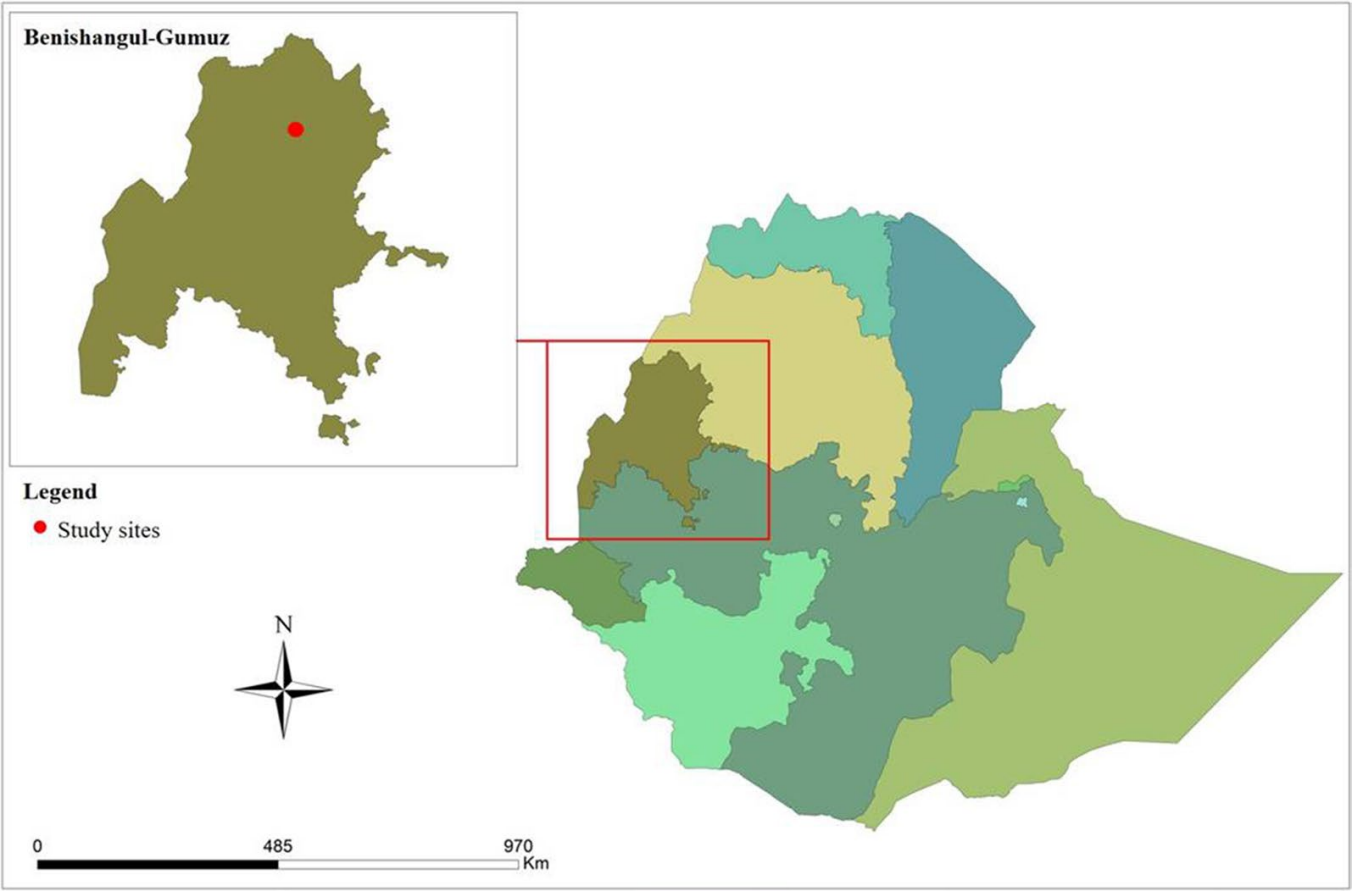
Map of districts in Ethiopia illustrating the location of Benshangul-Gumuz and the location of the sites in this study (red circle). Map produced using ArcMap v. 10.5

The study was conducted on eight farms in Dangur district (*woreda*), in Metekel zone, Benishangul-Gumuz region, Ethiopia (Fig. 1). Four of these farms were large-scale farms (larger than 100 hectares [ha]) and four were small scale farms (less than 100 ha). The farms cultivated a range of crops, including sesame, green grams, cow peas, and sorghum. The area has a single rainy season beginning in May that continues until October. The altitudes of the farms range between 751 and 1155m above sea level. The worker housing on these farms was generally of poor quality, consisting of wooden framed houses with grass or iron sheeting walls.

All data were collected between July and December 2017. The managers of two farms (one small, one large) where mosquito collections were made in July did not wish to continue in the following months, so these farms were replaced with two other farms based on their similarity in location, size and proximity to the breeding habitats with the previous ones. At each farm, two shelters were chosen, and collections were made inside and outside these shelters during each collection. One collection was made in each site each month, resulting in 16 indoor and 16 outdoor collections made each month. The collections were made between July and December (6 months), resulting in a total of 96 indoor and 96 outdoor collections.

### Mosquito collection

Mosquitoes were collected through human landing collection (HLC), the current gold standard for measurement of human biting activities of mosquitoes and entomological inoculation rates [10–12]. HLCs were chosen to have a collection method that reliably estimated human-vector contact as well as the necessity of a single method that could be used indoors, outdoors, and in night-time agricultural field work sites. HLCs involved the collection of mosquitoes on humans sitting with legs exposed during the collection hours. Each collector was provided with a flashlight, an aspirator to catch biting mosquitoes and one netting-topped polystyrene cup for each 1-hour catch-session. The mosquito holding cups were labelled with the name of the farm, shelter number, time-session and site of collection. These human collectors caught mosquitoes attempting to bite their exposed legs, and kept the mosquitoes sorted by hour of collection so that biting times could be determined. One collector sat indoors and the other one sat outdoors of the same shelter collecting the mosquitoes. The indoor and outdoor collectors exchanged site every hour to reduce biases due to differential attractiveness of the collectors to the mosquitoes. The collectors who conducted human landing collections were locally hired and trained. No personal information was collected about the collectors. The collectors were provided with prophylaxis (mefloquine [Lariam®]) to protect them from getting malaria or treated if they were diagnosed with malaria [13]. The collections were conducted over the course of 12 h (18.00–06.00 h).

Collections were made at the worker shelter camps in each farm which were inhabited by the workers.

Additionally, outdoor human landing collections were conducted in sites next to workers involved in night time work activities in the fields, such as the harvesting of sesame pods. One human landing collection was made in each farm between the months of September and December 2017, resulting in a total of 32 outdoor collections in the fields. Mosquitoes were killed and identified morphologically using appropriate identification keys [13]. They were then stored in 1.5ml Eppendorf tubes with silica gel before laboratory analysis.

### Laboratory analysis of mosquitoes

Almost all of the mosquitoes morphologically identified as belonging to the *Anopheles gambiae* complex were identified using standard polymerase chain reaction (PCR) to determine the species [14]. In brief, genomic DNA was mixed with the following primers in a 25 µL reaction: AR (5′-AAGTGTCCTTCTCCATCCRA-3′; specific for *An. arabiensis*), AG (5′ CTGGTTTGGTCGGCACGTTT-3; specific for *An. gambiae s.s*.), QD-b (5′-AGTGTCCAATGTCTGTGAAG-3′; specific for *Anopheles quadriannulatus* species B or *Anopheles amharicus*) and UN (5′ GTGTGCCCCTTCCTCGATGT-3′; common for all species). Amplification reactions contained 1 µL of DNA, 1.5 mM MgCl2, 10 mM Tris–HCl (pH 8.4), 50 mM KCl, 0.1 % Triton X-100, 200 µM of dNTPs (Amersham, Buckinghamshire, UK), 25 pmol of primers AR, AG, QD-b and UN and 0.25 U of SilverStar DNA polymerase (Eurogentec, Seraing, Belgium). Amplified PCR products were visualized on 2 % agarose gels, stained with ethidium bromide. An *An. arabiensis* strain from the Sekoru colony, maintained at the Vector Biology and Control Research Unit, Tropical and Infectious Diseases Research Centre of Jimma University (Jimma, Ethiopia), was used as a positive control.

A subsample of mosquitoes was also analysed to determine whether sporozoites were present, using established methods [15]. Briefly, heads and thoraces of mosquitoes were separated from the abdomen and were homogenized. ELISA plates were coated with a capture monoclonal antibody. Following aspiration to remove un-adsorbed capture antibody, plates were incubated with blocking buffer to prevent non-specific binding in subsequent steps. The blocking buffer was removed by aspiration and the mosquito homogenate was added to the plates. After 2 hours, samples were aspirated and horseradish peroxidase-linked monoclonal antibody was added. This was then aspirated before the peroxidase substrate solution, ABTS, was added. Absorbance values at 405nm were obtained 30–60 minutes later using an ELISA plate reader. Positive reactions were those with an absorbance value of greater than two times the average absorbance values of negative control samples.

### Behaviour‐adjusted patterns of human exposure

To understand the risk to humans for infective mosquito bites indoors and outdoors, the biting times of *An. arabiensis* were compared with the outdoor and indoor times of humans as collected by Tadesse et al. [16]. The proportion of the population indoors each hour was multiplied by the number of mosquitoes collected indoors to estimate the numbers of bites that would have occurred in the absence of any personal protection measure such as LLINs. The same procedure was repeated for mosquitoes biting outdoors. The sum of all hours represented the number of *An. arabiensis* bites one person might expect to receive. To estimate the exposure that one person using a LLINs between the hours of 21:00 and 6:00 h would receive, the number of mosquito bites expected each hour was multiplied by 0.063, a figure used by Seyoum et al. [17] (derived from two studies in Tanzania) to estimate the number of bites that would be received, even when using a net.

### Data analysis

Data were entered into Excel (Microsoft office 2007) for cleaning and data summary. Count data were analysed using generalized linear mixed effects models (glmer – function) [18] with R statistical software version 2.14.2 including the contributing packages MASS, lme4, glht, multcomp (alpha = 0.05) [19]. The biting time was included in the model as a fixed factor and collection round and site were included as random factors in the biting time analysis with Poisson distribution. The over-dispersion between data points that remained after adjustment for all other factors was adjusted by creating a random factor with a different level for each row of the data set.

The parameter estimates of the models were used to predict the mean counts or mean proportions and 95 % confidence intervals (CI) for the different size farms by removing the intercept from the models [19]. Multiple comparisons of treatments were also calculated based on the model parameter estimates.

The entomological inoculation rate was calculated as the product of the sporozoite rate and the mean number of *An. arabiensis* collected per person per night.

## Results

### Mosquito identification and analysis

Over the course of the 6 months of collection, 2829 mosquitoes were collected. Of these 1,970 (70 %) were *Anopheles* mosquitoes. Of the *Anopheles* mosquitoes collected, 1733 (88 %) were *Anopheles gambiae sensu lato* (*s.l*.). Other *Anopheles* species collected were *Anopheles pharoensis, Anopheles coustani, Anopheles demeilloni, Anopheles squamosus, Anopheles pretoriensis, Anopheles natalensis* and *Anopheles christyi* (see Table 1).

**Table 1:** Sporozoite ELISA results for *Anopheles* mosquito species collected from July to December 2017 using HLC in Dangur, Ethiopia

Species	Number tested	Number *Plasmodium falciparum* positive	Number *Plasmodium vivax (*Pv210) positive
*An. gambiae s.l.*	1702	8 (0.47 %)	1 (0.06 %)
*An. coustani*	80	0	0
*An. pharoensis*	42	0	0
*An. demeilloni*	87	0	0
*An. pretoriensis*	16	0	0
*An. natalensis*	2	0	0
Total	1929	8 (0.41 %)	1 (0.05 %)

Of the mosquitoes morphologically identified as *An. gambiae s.l*., 120 specimens were randomly selected and tested for species identification. Of the 120 tested, the DNAs of 117 were successfully amplified, and all of these were *An. arabiensis*. Hereafter, *An. gambiae s.l.* mosquitoes are referred to as *An. arabiensis*.

**Fig. 2:**
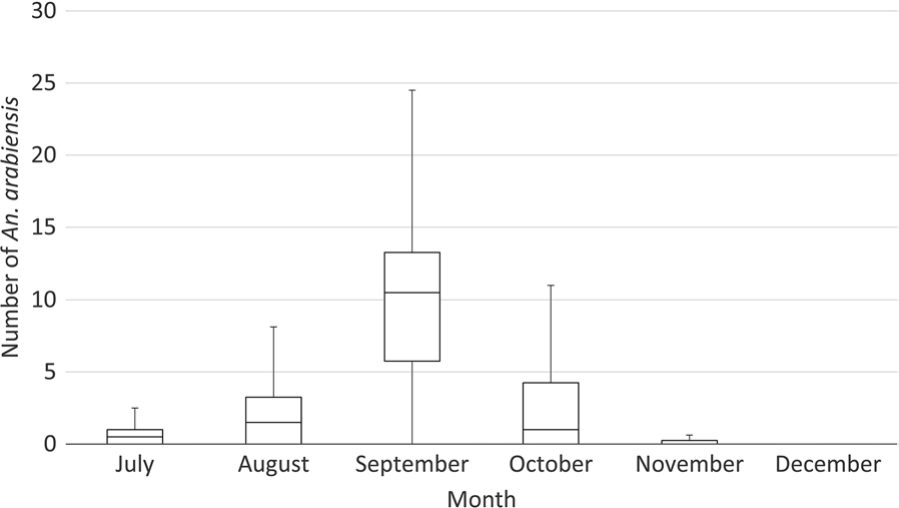
Tukey box-plots showing median and interquartile ranges of the number of *An. arabiensis* collected in indoor and outdoor human landing catches (n = 96) at workers’ shelters on small farms (< 100 ha), Dangur woreda, Ethiopia, 2017

**Fig. 3:**
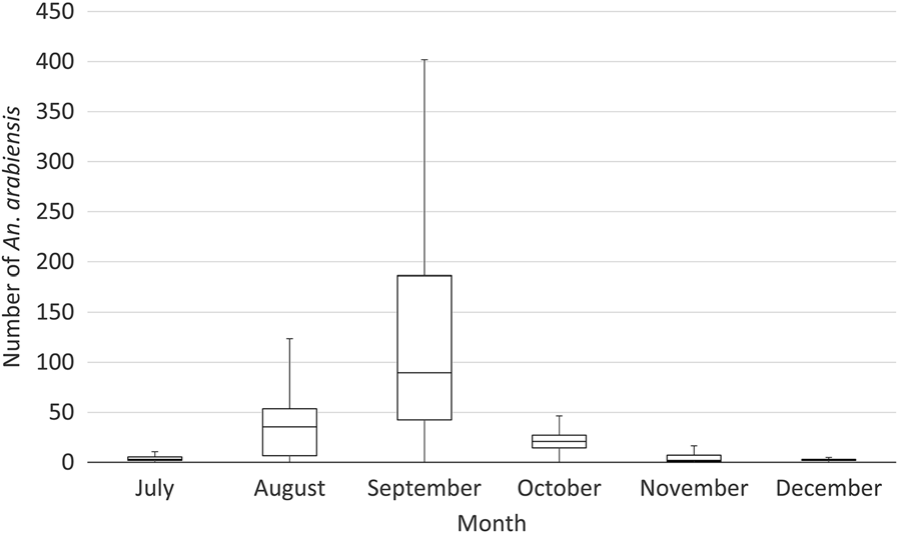
Tukey box-plots showing median and interquartile ranges of the number of *An. arabiensis* collected in indoor and outdoor human landing catches (n = 96) at workers’ shelters on large farms (> 100 ha), Dangur woreda, Ethiopia, 2017

The numbers of *An. arabiensis* collected increased from July, peaking in September, before decreasing to low levels in December. *Anopheles arabiensis* that were collected in large farms were ten times those collected in small farms (23.8 (95 %CI:17.1–60.5) and 2.2 (95 %CI:1.63-6.0), respectively; p < 0.001) (Figs. 2 and 3). *Anopheles arabiensis* that were collected in HLCs in fields (mean 2.0 per night, 95 %CI: 0-3.06) where night work was taking place were 1/5th of the outdoor collections made in the same months near workers’ shelters (10.1 (95 %CI: 4.45–15.7; *p* < 0.001)).

### Biting times and locations

**Fig. 4:**
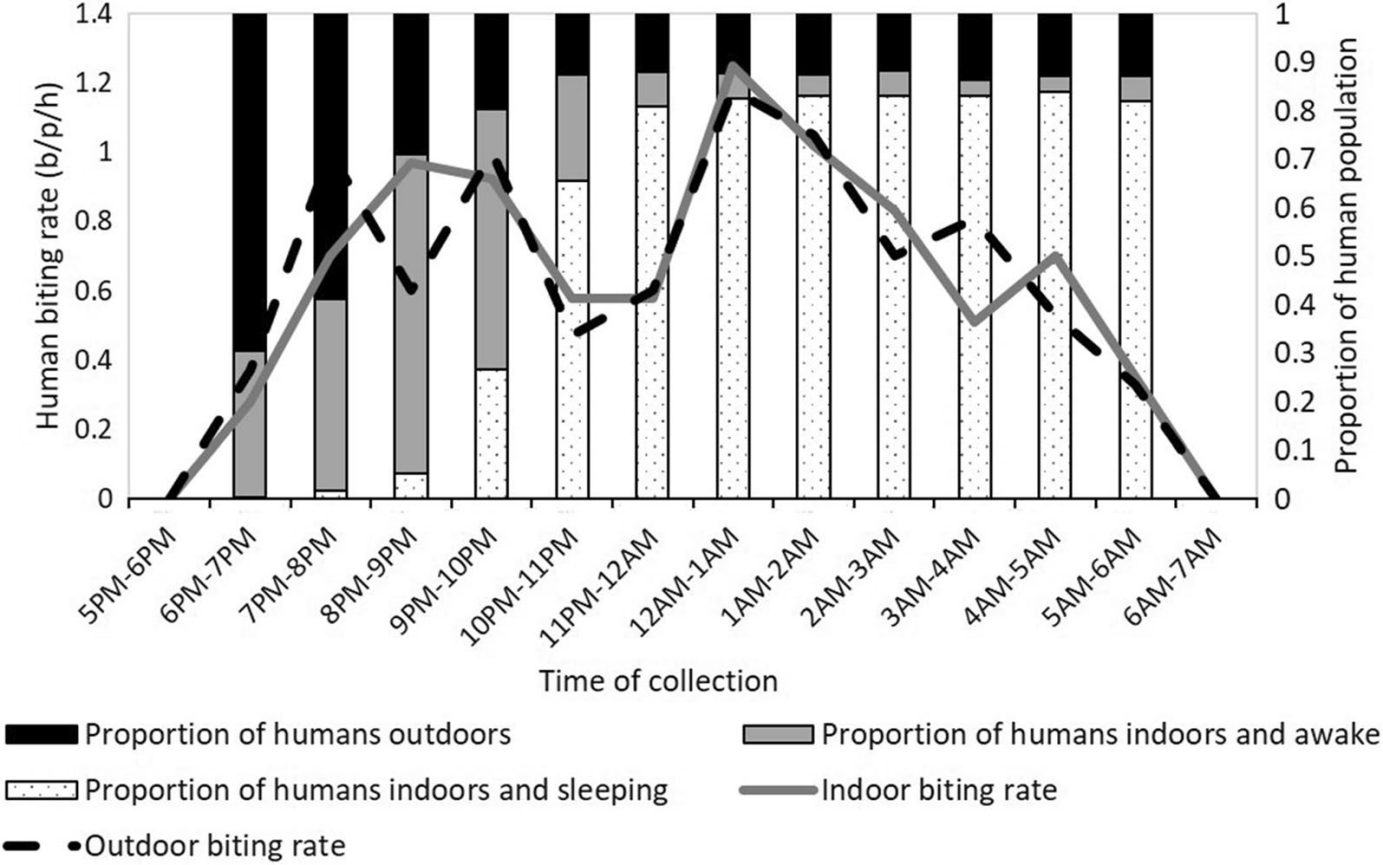
Directly measured hourly biting rate (bites per person per hour) indoors (solid grey line) and outdoors (dashed black line) in worker camps in eight farms in Dangur district (96 indoor collections, 96 outdoor collections) show 48 % of directly measured biting occurred indoors. Lines representing human biting rates are overlayed on top of bar charts representing the mean proportion of humans outdoors, indoors and awake, and indoors and asleep each hour of the evening. Approach derived from Monroe et al. [20]

*Anopheles arabiensis* were collected throughout the night in the study area near workers’ shelters with the highest numbers collected at 21:00–22:00 and 0:00–2:00 (Fig. 4). The biting times in the fields followed a similar pattern. The mean number of *An. arabiensis* collected indoors (8.7 (95 % CI: 4.6–12.9) per person per night) was similar to the number collected outdoors (8.6 (95 % CI: 4.7–12.6; p = 0.61) over the six months of collection.

### Sporozoite rates

In total, 1,929 female mosquitoes were tested for the *Plasmodium* circumsporozoite protein. The only species that tested positive was *An. arabiensis* (1,702 tested). Of these, 8 (0.47 %) tested positive for *P. falciparum*, while only 1 (0.06 %) was positive for *P. vivax* 210 (Table 1). No mosquito tested positive for *P. vivax* 247. The overall *Plasmodium* (any species) sporozoite rate was 0.53 %.

The entomological inoculation rate is based on the number of *An. arabiensis* bites one person would receive in one night. In this case, due to the similarity of indoor and outdoor biting rates (see above), the mean of the two rates (not including collections from the fields) was used (8.69). The overall *P.* sporozoite rate (*P. falciparum* and *P. vivax*) was 0.53 %. Therefore, the monthly entomological inoculation rate (EIR) of *An. arabiensis* in the living areas on the farms during the study period was 1.41 infectious bites per person per month (0.0053 sporozoite rate x 8.69 bites per night x 184days ÷ 6 months).

### Behaviour‐adjusted patterns of human exposure

As reported in a linked paper Tadesse et al. [16], the majority of people were outdoors in the early evening. The proportion of people outdoors decreased from 18:00 h to 22:00 h, after which only a small proportion of the population remained outdoors. As a result, despite the near equality of the numbers of *An. arabiensis* that were collected indoors and outdoors, the risk for actual bites from *An. arabiensis* was greatest indoors at night, between 20:00 h and 5:00 h. The risk outdoors was considerably lower but was greatest from 19:00 h-20:00 h (Fig. 4).

**Fig. 5:**
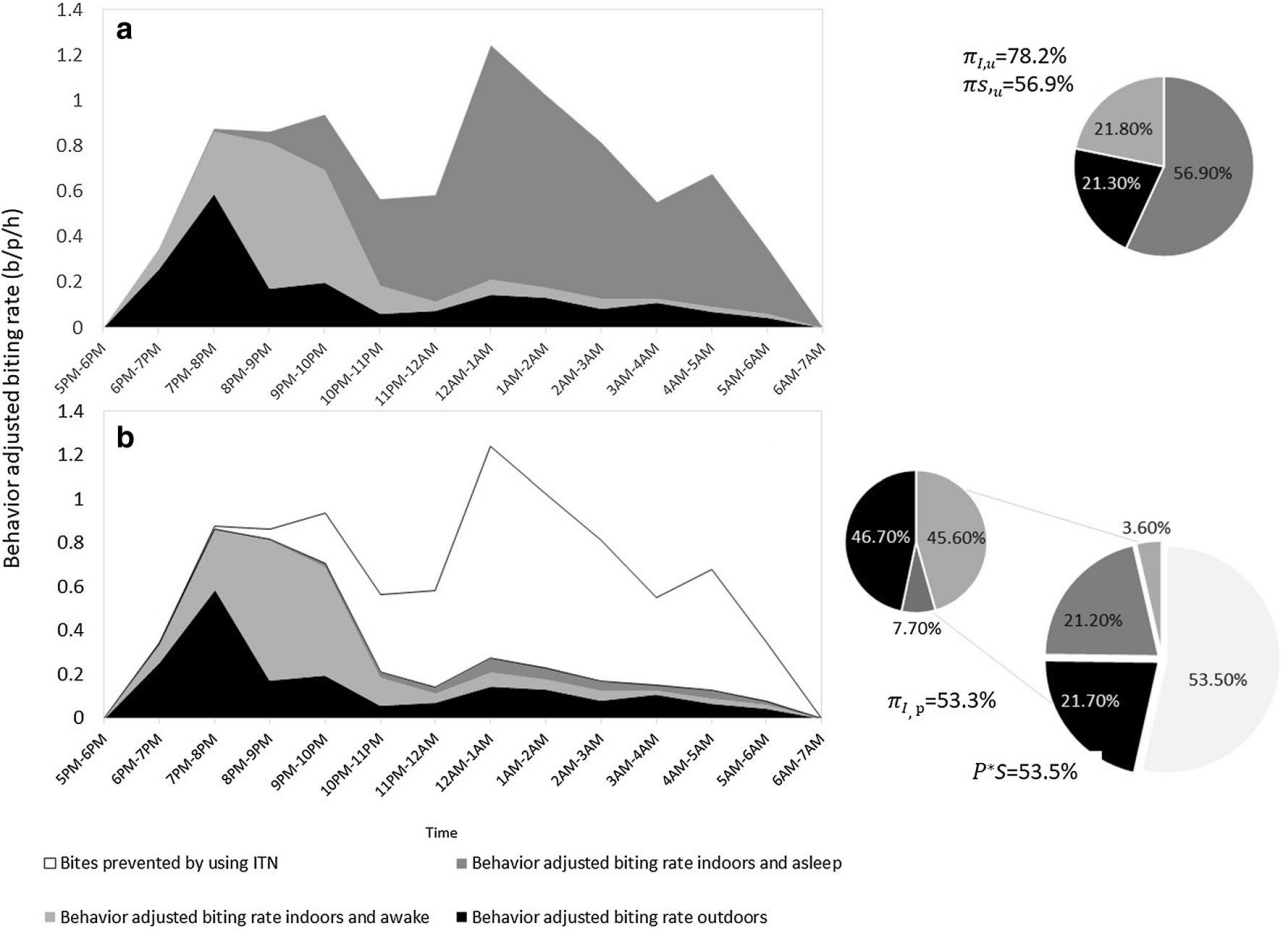
Human and *An. arabiensis* data were combined to derive human adjusted biting rates for ITN unprotected and protected individuals. (A) Behaviour-adjusted biting rates per person per hour for an unprotected individual are presented. The percentage of vector bites occurring indoors for unprotected individuals is 75 %, and the percentage of bites occurring while asleep indoors for an unprotected individual is 54 %. (B) Behaviour-adjusted estimates of *An. arabiensis* bites per hour per person in the study areas if ITNs were used, using the typical start and end times shown in the study area. The percentage of vector bites that would be prevented by using an ITN is 51 %. Approach and calculations derived from Monroe et al., 2020 [20]

When the indoor and outdoor behaviour of humans and mosquitoes were combined, the estimated total number of bites one might be expected to receive was 8.81 bites per person per night. When the actual behaviour of people working in the farms was calculated, including bed net use by a minority of workers, the estimated number of bites per person per night was calculated as 7.41. If one used a bed net between the hours of 21:00 h and 6:00 h (the hours during which most people who had bed nets used bed nets), this number would decrease to 2.49, over three and a half times less than an unprotected individual (Fig. 5).

## Discussion

The mosquito literature from Benishangul-Gumuz is extremely limited and no literature related to the malaria vectors in Dangur woreda was found. In this study, eight species of *Anopheles* were collected and identified, with *An. arabiensis*, the major malaria vector in Ethiopia, as the most prevalent. Additionally, *An. arabiensis* was the only species found to have *Plasmodium* circumsporozoite protein in the head and thorax, indicating that this is likely to be the most important vector in agricultural areas in Dangur woreda. However, *An. coustani* and *An. pharoensis* were also collected in small numbers and these species have been found to be capable of transmitting *Plasmodium* parasites in laboratory and field studies [21, 22].

The biting times of *An. arabiensis* present a challenge for the protection of migrant workers from infectious bites. *An. arabiensis* were found biting indoors and outdoors at nearly all time points, indicating an equal risk for workers staying indoors and outdoors, similar to elsewhere in Ethiopia [23–26]. Pooling the overall *Anopheles* species collection, Kenea et al. [26] reported that the outdoor density was 3.3 times higher than the indoor density which also indicates the importance of outdoor biting activities for malaria transmission. Furthermore, biting started as early as the 18:00 h-19:00 h time period and reached a first peak between 19:00 and 22:00. Early biting of *An. arabiensis* has been found in other studies in Ethiopia, but not always with a second peak [27]. Tadesse et al. [16] found that at least half of workers went to bed by 21:30, but this would leave them exposed to the first peak for 2.5 hours before sleep. Additionally, LLIN use by migrant workers was almost none, as they often left their nets with their families when they came to the farms for work and the farm owners do not provide any [16]. One limitation of this study is that the HLCs were completed by 6:00 h, whereas mosquito host-seeking may have continued on beyond this time point, posing further risk for workers in the early morning. Higher mosquito densities in the large farms suggest that more farming activities and hence higher workers’ population might enhance the population of the vectors in those farms. Janko et al. [22] reported a positive correlation of agriculture coverage and the density of biting *An. gambiae s.l.* For example, they indicated that a 15 % increase in agricultural cover was associated with increased probabilities of *An. gambiae s.l.* biting indoors.

The lower mosquito vector density in the fields compared to outdoors near shelters might be due to higher attraction to places where the host population was higher [28, 29].

Seasonal variations were observed in the numbers of mosquitoes collected that were generally similar to those in other areas in Ethiopia, with an increase in mosquito populations during the main rainy season, and a decrease in population size as the rains end towards the end of the year [23, 24]. It is important to notice that the peak of the agricultural activities and hence demand for workers occurs during the time of the year with the largest populations of *An. arabiensis*.

Finally, the detection of sporozoites in *An. arabiensis* allowed us to calculate their entomological inoculation rate. This was found to be 1.41 infectious bites per month in the study area during the 6 months that monitoring was conducted. This figure is similar to EIR found in other parts of Ethiopia. Massebo et al. [24] found a yearly EIR of 17.1 in Chano, in the Southern Nations, Nationalities, and People’s Region. In both locations annual EIR is well above 0.003 inoculations per night, the threshold below which Smith et al. [25] estimated EIRs needed to drop below for substantial reductions of malaria prevalence. However, EIRs in highly malarious areas where vector control has had an impact can often reach much higher levels [30], indicating that improved vector control and treatment for malaria could have an important impact on transmission in these farms. Therefore, it might be very useful for the national malaria control strategy to focus on universal coverage of bed net distribution, including for the mobile and migrant populations and improvement of other vector control measures such as better IRS and environmental management (destruction of potential aquatic habitats) in such areas of the country.

Insecticide resistance was not assessed as a part of this study. Further work should be done to evaluate the insecticide susceptibility of *An. arabiensis* in this area. As IRS is not currently well implemented for protection of many of the workers due to the poor quality of structures or incomplete walls, the susceptibility testing should focus on the evaluation of Actellic 300CS, pyrethroids, Chlorfenapyr, and the synergism of pyrethroid susceptibility with piperonyl butoxide.

There were some limitations to the current study. Collections were only made over a six-month period, and not over the whole year. While the six months chosen were the primary season for malaria transmission, there may be risks of malaria transmission outside these months. Also, collections in the fields were conducted from September until December, whereas collections in the worker’s shelter areas were conducted from July to December. An additional limitation of this study was that the mosquito collections ended at 06:00, and there may be some mosquito biting after this time. The ELISA reactions were considered positive at twice the value of the mean optical density of negative controls and were not re-boiled when positive. Finally, the current recommendations for analysis of human exposure [20] do not take into account mosquito response to human behaviour. Outdoor biting mosquitoes that do not find humans outdoor may to move indoors to feed on humans, and thus we may have underestimated the risk, for both net users, and, to a greater extent, non-users.

## Conclusions

*Anopheles arabiensis* is the most likely primary malaria vector in the agricultural development areas Dangur District like the other malarious parts of the country. While the EIRs do not indicate high rates of transmission, the low use of vector control interventions and lack of access for treatment result in a real risk of malaria for migrant workers staying in these locations. Improved malaria prevention and treatment could have a valuable impact on worker health and productivity in these areas. In addition, the results of this study indicated that proper bed net use between 21.00 h and 6.00 h could reduce an estimated number of bites per person per night by about three-fold. However, it seems likely that the behaviour of *An. arabiensis*, particularly outdoor biting and a wide range of biting times will also pose challenges to implementing effective vector control.

## Data Availability

The data generated and/or analysed during the current study are available at Abt Associates Inc.

## Abbreviations

ABTS: 2,2’-azino-bis(3-ethylbenzothiazoline-6-sulfonic acid).
ADLI: Agricultural Development Led Industrialization.
CI: Confidence interval.
DNA: Deoxyribonucleic acid.
EIR: Entomologic inoculation rate.
ELISA: Enzyme-linked immunosorbant assay.
HLC: Human landing catch.
IRS: Indoor residual spray.
LLIN: Long-lasting insecticidal net.
PCR: Polymerase chain reaction.

## References

[1] World Malaria Report. 2020.Geneva: World Health Organization; 2020.

[2] Bhatt S, Weiss DJ, Cameron E, Bisanzio D, Mappin B, Dalrymple U (2015). **The effect of malaria control on***Plasmodium falciparum***in Africa between 2000 and 2015**. Nature.

[3] Noor AM, Kinyoki DK, Mundia CW, Kabaria CW, Mutua JW, Alegana VA (2014). **The changing risk of***Plasmodium falciparum***malaria infection in Africa: 2000–10: a spatial and temporal analysis of transmission intensity**. Lancet.

[4] WHO, UNICEF. Achieving the malaria Millennium Development Goal target: reversing the incidence of malaria 2000–**2015**. Geneva, World Health Organization; 2015. http://apps.who.int/iris/bitstream/10665/184521/1/9789241509442_eng.pdf. Accessed 30 Jan 2021.

[5] Federal Democratic Republic of Ethiopia Ministry of Health. National Malaria Elimination Roadmap. Addis Ababa, 2016.

[6] World Bank Group (2016). Federal Democratic Republic of Ethiopia, priorities for ending extreme poverty and promoting shared prosperity, systematic country diagnostic.

[7] Argaw D, Mulugeta A, Herrero M, Nombela N, Teklu T, Tefera T (2013). Risk factors for visceral leishmaniasis among residents and migrants in Kafta-Humera, Ethiopia. PLoS Neglect Trop Dis.

[8] Alemu K, Worku A, Berhane Y, Kumie A (2014). Men travelling away from home are more likely to bring malaria into high altitude villages, northwest Ethiopia. PLoS One.

[9] Braack L, Hunt R, Koekemoer LL, Gericke A, Munhenga G (2015). Biting behaviour of African malaria vectors: 1. Where do the main vector species bite on the human body?. Parasit Vectors.

[10] Magbity EB, Magbity EB, Lines JD, Marbiah MT, David K, Peterson E (2002). How reliable are light traps in estimating biting rates of adult *Anopheles gambiae s.l.* (Diptera: Culicidae) in the presence of treated bed nets?. Bull Entomol Res.

[11] Briet OJT, Chitnis N (2013). Effects of changing mosquito host searching behaviour on the cost effectiveness of a mass distribution of long-lasting, insecticidal nets: a modelling study. Malar J.

[12] Lima JB, Rosa-Freitas MG, Rodovalho CM, Santos F, Lourenco-de-Oliveira R (2014). Is there an efficient trap or collection method for sampling *Anopheles darlingi* and other malaria vectors that can describe the essential parameters affecting transmission dynamics as effectively as human landing catches? A review. Mem Inst Oswaldo Cruz.

[13] Gillies MT, Coetzee M (1987). A Supplement to the Anophelinae of Africa South of the Sahara (Afrotropical Region).

[14] Scott JA, Brogdon WG, Collins FH (1993). Identification of single specimens of the *Anopheles gambiae* complex by the polymerase chain reaction. Am J Trop Med Hyg.

[15] Wirtz RA, Charoenvit Y, Campbell GH, Burkot tr, Schneider I, Esser KM (1987). Comparative testing of monoclonal antibodies against *Plasmodium falciparum* sporozoites for ELISA development. Bull World Health Organ.

[16] Yehualashet T, Irish SR, Chibsa S, Dugassa S, Lorenz LM, Gebreyohannes A. et al. Malaria prevention and treatment in migrant agricultural workers in Dangur district, Benishangul-Gumuz, Ethiopia: social and behavioral aspects. Malar J. 2021 (in press). 10.1186/s12936-021-03766-3 PMC8135166 34011347

[17] Seyoum A, Sikaala CH, Chanda J, Chinula D, Ntamatungiro AJ, Hawela M (2012). Human exposure to anopheline mosquitoes occurs primarily indoors, even for users of insecticide-treated nets in Luangwa Valley, South-east Zambia. Parasit Vectors.

[18] Seavy NE, Quader S, Alexander JD, Ralph CJ. Generalized linear models and point count data: statistical considerations for the design and analysis of monitoring studies.In: Ralph CJ, Rich TD, eds. Bird conservation implementation and integration in the Americas. Albany: USDA Forest Service General Technical Report PSW; 2005.

[19] R Development Core Team (2014). R: A language and environment for statistical computing.

[20] Monroe A, Moore S, Okumu F, Kiware S, Lobo NF, Koenker H (2020). Methods and indicators for measuring patterns of human exposure to malaria vectors. Malar J.

[21] Abt Associates Inc. Report. The PMI Africa IRS (AIRS) Project Indoor Residual Spraying (IRS) task order four & indoor residual spraying task order six. 2015.

[22] Janko MM, Irish SR, Reich BJ, Peterson M, Doctor SM, Mwandagalirwa MK (2018). **The links between agriculture**, *Anopheles***mosquitoes, and malaria risk in children younger than 5 years in the Democratic Republic of the Congo: a population-based, cross-sectional, spatial study**. Lancet Planet Health.

[23] Yohannes M, Boelee E (2012). Early biting rhythm in the Afro-tropical vector of malaria, *Anopheles arabiensis*, and challenges for its control in Ethiopia. Med Vet Entomol.

[24] Massebo F, Balkew M, Gebre-Michael T, Lindtjørn B (2013). Entomologic inoculation rates of *Anopheles arabiensis* in Southwestern Ethiopia. Am J Trop Med Hyg.

[25] Smith T, Maire N, Dietz K, Killeen GF, Vounatsou P, Molineaux L (2006). Relationship between the entomologic inoculation rate and the force of infection for *Plasmodium falciparum* malaria. Am. J Trop Med Hyg.

[26] Kenea O, Balkew M, Tekie H, Gebre-Michael T, Deressa W, Loha E (2016). Human-biting activities of *Anopheles* species in south-central Ethiopia. Parasit Vectors.

[27] Yohannes M, Boelee E (2012). Early biting rhythm in the Afro-tropical vector of malaria, *Anopheles arabiensis*, and challenges for its control in Ethiopia. Med Vet Entomol.

[28] Debebe Y, Hill SR, Tekie H, Dugassa S, Hopkins RJ, Ignell R (2020). Malaria hotspots explained from the perspective of ecological theory underlying insect foraging. Sci Rep.

[29] Kaindoa EW, Mkandawile G, Ligamba G, Kelly-Hope LA, Okumu FO (2016). Correlations between household occupancy and malaria vector biting risk in rural Tanzanian villages: implications for high-resolution spatial targeting of control interventions. Malar J.

[30] Kilama M, Smith DL, Hutchinson R, Kigozi R, Yeka A, Lavoy G (2014). Estimating the annual entomological inoculation rate for *Plasmodium falciparum* transmitted by *Anopheles gambiae s.l.* using three sampling methods in three sites in Uganda. Malar J.

